# Reply: Request for clarification on symptom assessment methodology in high‐risk population colonoscopy study

**DOI:** 10.1002/cam4.6164

**Published:** 2023-06-02

**Authors:** Mingqing Zhang, Yongdan Zhang, Wen Zhang, Lizhong Zhao, Haoren Jing, Xiaojing Wu, Lu Guo, Haixiang Zhang, Yong Zhang, Siwei Zhu, Shiwu Zhang, Xipeng Zhang

**Affiliations:** ^1^ Nankai University School of Medicine Nankai University Tianjin China; ^2^ Department of Colorectal Surgery Tianjin Union Medical Center Tianjin China; ^3^ Tianjin Institute of Coloproctology Tianjin China; ^4^ The Institute of Translational Medicine Tianjin Union Medical Center of Nankai University Tianjin China; ^5^ Center for Applied Mathematics Tianjin University Tianjin China; ^6^ Department of Pathology Tianjin Union Medical Center Tianjin China

We would like to express our gratitude to Safari, Farzad, and Noursina, Ali, for their insightful comments about our article regarding the questionnaire procedure and the assessment of symptoms, including a history of chronic constipation, chronic diarrhea, and mucous blood stools.

CRC screening has been carried out in China since the 1970s. The High‐Risk Factor Questionnaire (HRFQ) for CRC screening in China was developed based on the CRC General Census conducted twice in Jiashan County and Haining County, Zhejiang Province. This questionnaire has been included in national screening programs since 2007 and has been used in many cities in China.^1^, ^2^, ^3^ Since 2012, CRC screening in community allied third‐grade class‐A hospitals has been initiated in Tianjin City, China.

First, our screening protocol was developed by the Tianjin CRC Screening Office and conducted in primary care units and medical institutions in Tianjin that perform colonoscopies. Participants identified as high risk were suggested to undergo colonoscopy at designated hospitals through advice notes issued by the screening physicians. Moreover, HRFQ, FIT, and colonoscopy are free and performed at CRC screening units.

As for the assessment of symptoms, our HRFQ includes a history of chronic constipation, chronic diarrhea, and mucous bloody stools. These three past symptoms were considered along with a history of adverse life events (e.g., divorce, death of a close relative, etc.), a history of chronic appendicitis or appendectomy, and a history of chronic cholecystitis or gallstones, and the presence of two or more of these was considered as one of the criteria for identifying a high‐risk group. Chronic diarrhea is defined in this questionnaire as diarrhea lasting cumulatively more than 3 months in the last 2 years, with each episode lasting more than 1 week; chronic constipation is defined as constipation for more than 2 months per year in the last 2 years. Mucus bloody stools were self‐reported by the participants. As stated in the manuscript, only 39.41% of the screening population completed a colonoscopy within 1 month, so those with symptoms at the time of initial screening were advised to seek a definitive diagnosis promptly and were excluded from screening. The data from the screening cohort also revealed that the rate of FIT positivity was lower in individuals with a self‐reported history of mucous bloody stools than in those without such a history, and the rate of FIT positivity would likely be higher if symptoms were present at the time of screening (Table 1).

**TABLE 1:** Comparison of FIT results among patients with or without a history of mucous bloody stools.

	History of mucous bloody stools	No history of mucous bloody stools	Chi‐squared test	*p*
Positive FIT	3412 (41.21)	30,057 (72.37)	3042.022	<0.001
Negative FIT	4868 (58.79)	11,473 (27.63)		

The reviewer's point is valid and significant. Our search for keywords “asymptomatic” and “colorectal screening” revealed that some of the inclusion and exclusion criteria for screening in asymptomatic individuals did not consider the relevant history of three symptoms.^4^, ^5^, ^6^, ^7^, ^8^ Other articles we found used descriptions related to asymptomatic status, such as reporting no visible rectal bleeding, no change in bowel habits, and no recent or current lower abdominal pain,^9^ or no abdominal pain, diarrhea, or hematochezia.^10^ Our HRFQ, a validated screening tool, considers mucus bloody stools, chronic diarrhea, and chronic constipation as intermittent history over time. Although these three symptoms were present as a past history, we collectively agreed to remove the term “asymptomatic” from the manuscript description considering other articles on screening.

We also evaluated the risk factors documented in the HRFQ. We found that a history of chronic constipation, chronic diarrhea, and mucous bloody stools were not high‐risk factors for colorectal neoplasia but may improve patient compliance. We advocate that a history of these symptoms, albeit not currently present, is a self‐assessment made by the patients based on their own experiences, indicating their awareness of their health status. Regardless of whether patients can accurately assess their symptoms, notifying their healthcare provider of symptoms such as chronic constipation, chronic diarrhea, and mucoid stools could indicate that they are attentive to changes in their GI tract that could be linked to underlying health issues. By doing so, patients increase their chances of receiving timely and effective medical attention, which may help detect and address medical conditions sooner rather than later.

## AUTHOR CONTRIBUTIONS

**Mingqing Zhang:** Writing – original draft (equal). **Yongdan Zhang:** Writing – original draft (equal). **Wen Zhang:** Validation (equal). **Lizhong Zhao:** Validation (equal). **Haoren Jing:** Validation (equal). **Xiaojing Wu:** Validation (equal). **Lu Guo:** Validation (equal). **Haixiang Zhang:** Validation (equal). **Yong Zhang:** Validation (equal). **Siwei Zhu:** Writing – review and editing (equal). **Shiwu Zhang:** Writing – review and editing (equal). **Xipeng Zhang:** Writing – review and editing (equal).

## CONFLICT OF INTEREST STATEMENT

The authors disclose no conflicts.

## Data Availability

Data sharing is not applicable to this article as no new data were created or analyzed in this study.
